# Efficacy and safety of anlotinib combined with PD‐1/PD‐L1 inhibitors as second‐line and subsequent therapy in advanced small‐cell lung cancer

**DOI:** 10.1002/cam4.5360

**Published:** 2022-10-17

**Authors:** Lian Yu, Jianlin Xu, Rong Qiao, Baohui Han, Hua Zhong, Runbo Zhong

**Affiliations:** ^1^ Department of Pulmonary Medicine, Shanghai Chest Hospital Shanghai Jiaotong University Shanghai China

**Keywords:** anlotinib, antiangiogenics, PD‐1/PD‐L1, small‐cell lung cancer

## Abstract

**Objectives:**

Treatments for advanced small‐cell lung cancer (SCLC) patients who are resistant to first‐line chemotherapy are limited. Given that antiangiogenic agents and immune‐checkpoint inhibitors (ICIs) can confer synergistic therapeutic benefits, combination therapy should be considered. We explored the efficacy and safety of combination therapy with anlotinib and programmed cell death protein 1 (PD‐1)/programmed cell death‐ligand 1 (PD‐L1) inhibitors as second‐line and subsequent therapy for advanced SCLC.

**Materials and Methods:**

We reviewed advanced SCLC patients at Shanghai Chest Hospital who had received anlotinib in combination with ICIs from November 2016 to November 2020 as second‐ and subsequent‐line treatment. Patients with advanced SCLC who had received paclitaxel monotherapy as second‐line treatment were included as the control group.

**Results:**

A total of 141 patients were included in the final analysis (40 in the combination therapy group and 101 in the paclitaxel monotherapy group). The median progression‐free survival (PFS) times for the combination therapy and paclitaxel monotherapy groups were 3.40 and 2.83 months (*p* = 0.022), respectively, while the median overall survival (OS) times for the combination therapy and paclitaxel monotherapy groups were 8.20 and 5.87 months (*p* = 0.048), respectively. Hypertension and hepatic dysfunction were the most pronounced adverse events of combination therapy and two patients changed regimens due to severe fatigue and anorexia.

**Conclusion:**

The combination of anlotinib and PD‐1/PD‐L1 blockade has promising efficacy and safety as a second‐line or subsequent therapy for SCLC.

## Graphical abstract

Treatments for advanced small‐cell lung cancer (SCLC) patients who are resistant to first‐line chemotherapy are limited. Given that antiangiogenic agents and immune‐checkpoint inhibitors (ICIs) could confer synergistic therapeutic benefits, combination therapy should be considered. We explored the efficacy and safety of the multitargeted antiangiogenic agent anlotinib and PD‐1/ PD‐L1 inhibitors combination therapy as second‐line or subsequent therapy for advanced SCLC. Compared with paclitaxel monotherapy as second‐line treatment, the combination of anlotinib and PD‐1/PD‐L1 blockade has promising efficacy and safety, involving 50% of patients receiving this combination therapy as third‐line and above, even as an end‐stage treatment.

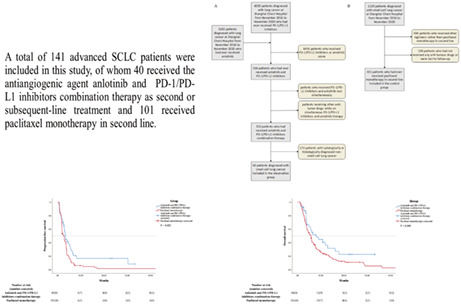

## 1 INTRODUCTION

Small‐cell lung cancer (SCLC) is a highly malignant and lethal tumor, accounting for 15% of all diagnosed lung cancers. Two thirds of SCLC patients present with extensive‐stage disease (ED).^1^, ^2^, ^3^ Despite being responsive to initial cytotoxic therapy, advanced SCLC is characterized by extensive metastasis, acquired therapeutic resistance, and early recurrence.^4^, ^5^

For over three decades, platinum‐etoposide chemotherapy, with an initial response rate of up to 78%, has remained as the first‐line standard of care for ED‐SCLC. However, median progression‐free survival (PFS) and median overall survival (OS) times for this standard frontline therapy are approximately 5 months and 10 months, respectively.^6^, ^7^, ^8^ Incorporation of immune‐checkpoint inhibitors (ICIs) in therapeutic options for SCLC has been hypothesized to improve patient outcomes. For instance, a combination of programmed cell death‐ligand 1 (PD‐L1) inhibitor with platinum‐etoposide chemotherapy as first‐line treatment options for ED‐SCLC prolongs the median OS by 2–3 months.^9^, ^10^ The second‐line and later‐line regimens for ED‐SCLC are limited. Regarding second‐line therapeutic options for ED‐SCLC, topotecan chemotherapy is the most common. However, its overall efficacy is far from satisfactory.^11^ Programmed cell death‐1 (PD‐1) inhibitor monotherapy, such as nivolumab and pembrolizumab, were used as third‐line and late‐line treatment options for ED‐SCLC; however, they had no significant survival benefits.^12^, ^13^

Advances in the use of antiangiogenic agents for treatment of ED‐SCLC had been painstakingly slow until the emergence of anlotinib, a novel multitargeted tyrosine kinase receptor inhibitor (TKI). Anlotinib targets vascular endothelial growth factor receptors (VEGFRs), fibroblast growth factor receptors (FGFRs), platelet‐derived growth factor receptors (PDGFRs), and stem cell factor receptors (c‐kit),^14^ thereby exerting inhibitory effects on tumor growth and angiogenesis.^15^, ^16^ Based on the findings of the clinical trial, ALTER 1202,^17^ anlotinib was approved and launched in China as a third‐line therapy for ED‐SCLC patients.

Antiangiogenic agents have the potential to reprogram the immunosuppressive tumor microenvironment and prompt tumor vessel normalization. Furthermore, a combination of anlotinib and a PD‐1 blockade promotes the infiltration of innate immune cells and confers potential synergistic antitumor activities.^18^ The synergistic antitumor activities of antiangiogenesis and immune checkpoint blockade have been shown to improve the treatment outcomes of various solid tumors in clinical trials.^19^, ^20^, ^21^, ^22^, ^23^, ^24^ We evaluated the efficacy and safety of anlotinib combined with PD‐1/PD‐L1 blockade therapy as second‐line and subsequent therapeutic options for advanced SCLC.

## 2 METHODS

### 2.1 Patients

**FIGURE 1:**
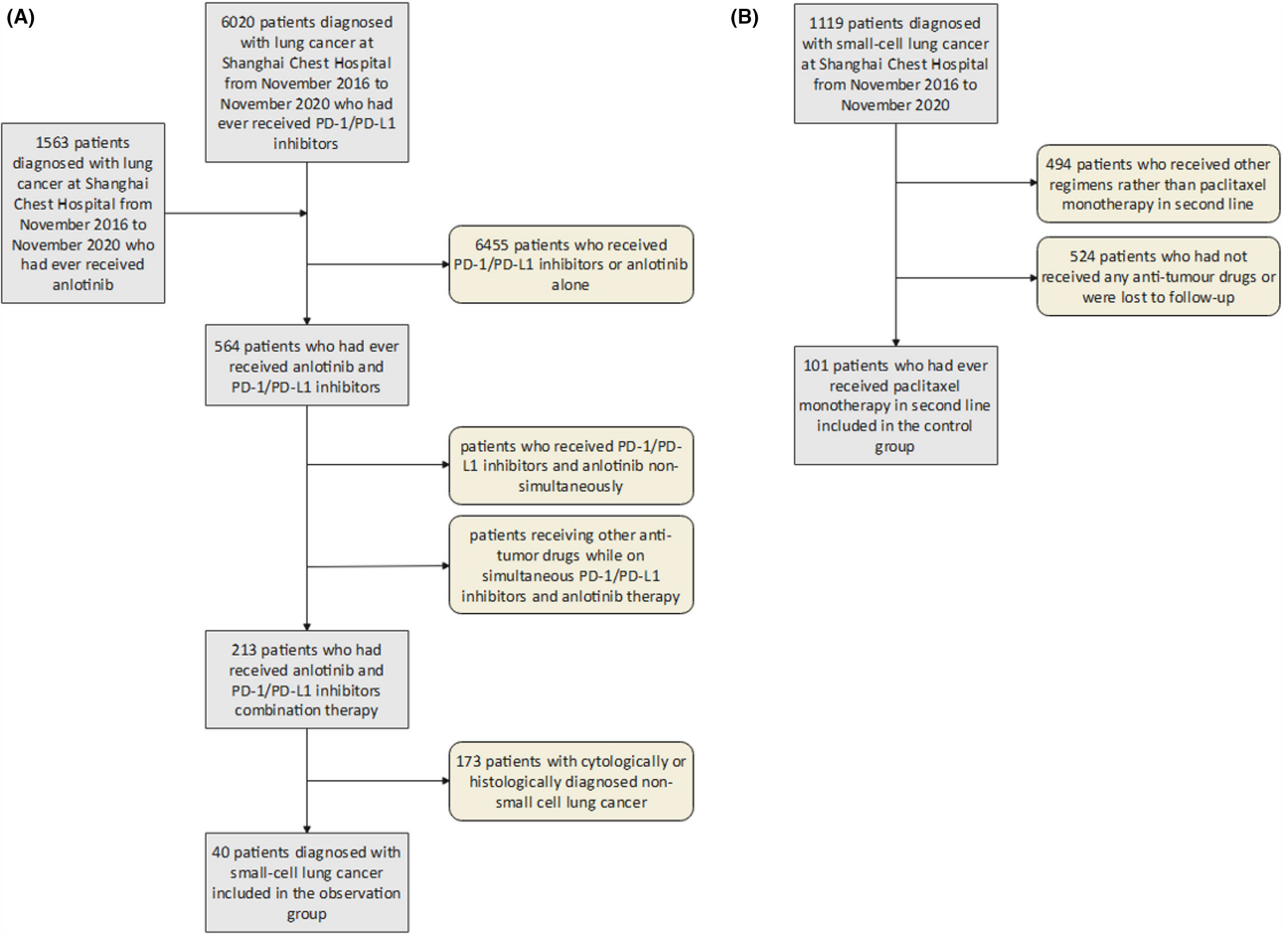
Flowchart showing patient screening of (A) the anlotinib and PD‐1/PD‐L1 inhibitors combination therapy group and (B) the paclitaxel monotherapy group.

We retrospectively reviewed the clinical data for advanced SCLC patients at Shanghai Chest Hospital from November 2016 to November 2020. The inclusion criteria were: (i) patients with histologically confirmed advanced SCLC; (ii) patients with disease progression after prior standard chemotherapy; and (iii) patients who received a combination of anlotinib and ICIs as second‐line or subsequent treatment options. Advanced SCLC patients who received paclitaxel monotherapy as second‐line treatment options were included as the control group. Patients with incomplete information were excluded (Figure 1).

This retrospective study was approved by the Ethical Committee of Shanghai Chest Hospital and was performed following the Helsinki Declaration of 1964 (revised 2008). The requirement for informed consent from the patients was waived.

### 2.2 Clinical assessments and follow‐up

Before treatment, all patients were restaged according to the 8th edition of the tumor, node, metastasis (TNM) classification system. During the therapeutic course, treatment efficacy was assessed by a chest computed tomography (CT) scan every 2 months, with additional abdominal ultrasound and cranial magnetic resonance imaging (MRI) as well as bone emission computed tomography (ECT) if necessary, until disease progression, termination of therapy or last follow‐up visit, whichever occurred first.

The best objective responses (BORs) were evaluated according to the Response Evaluation Criteria in Solid Tumors (RECIST v1.1). This includes complete response (CR), partial response (PR), stable disease (SD), or progressive disease (PD). Objective response rate (ORR) was defined as the proportion of patients with CR or PR, while disease control rate (DCR) was defined as the proportion of patients with CR or PR and SD. The PFS was calculated from the beginning of therapeutic administration to disease progression, regimen change, or last follow‐up visit, depending on which one occurred first. The OS was calculated from the beginning of therapeutic administration to death or last follow‐up visit. Adverse events (AEs) were categorized according to the Medical Dictionary for Regulatory Activities (MedDRA v20.0) and graded according to the Common Terminology Criteria for Adverse Events (CTCAE v5.0).

The main assessment indicators included PFS, OS, ORR, DCR, and AEs. The last follow‐up date for the study was September 26, 2021.

### 2.3 Statistical analysis

Statistical analyses were performed using the SPSS 26.0 statistical software (IBM, Armonk, NY, USA). Patient ages are presented as median and range, while the other patient characteristics were categorical variables, recorded as the number and percentage of patients involved. The BORs were compared using the chi‐square test. Survival (PFS and OS) outcomes were estimated by the Kaplan–Meier method and compared using the log‐rank test. Multivariate Cox regression was used to identify significantly different factors related to PFS and OS. Data are presented as median values with their respective 95% confidence intervals (CIs). *p ≤* 0.05 was considered statistically significant.

## 3 RESULTS

### 3.1 Baseline characteristics

A total of 141 patients were included in this study (40 patients in the observation group administered with anlotinib and PD‐1/PD‐L1 inhibitors as combination therapy for second‐line and subsequent treatment; 101 patients in the control group administered with paclitaxel monotherapy as second‐line therapy). The demographic information and treatments for patients in observation and control groups are shown in Table 1.

**TABLE 1:** Clinical characteristics of the group that received anlotinib and PD‐1/PD‐L1 inhibitors combination therapy and the group that received paclitaxel monotherapy

Characteristics	Anlotinib and PD‐1/PD‐L1 inhibitors combination therapy (*N* = 40) (%)	Paclitaxel monotherapy (*N* = 101) (%)
Age (years)		
<65	21 (52.5)	63 (62.4)
≥65	19 (47.5)	38 (37.6)
Median	64	60
Range	49–77	23–78
Gender		
Male	38 (95.0)	89 (88.1)
Female	2 (5.0)	12 (11.9)
Smoking status		
Never‐smoker	5 (12.5)	16 (15.8)
Smoker	35 (87.5)	85 (84.2)
Smoking index		
<600	14 (35.0)	42 (41.6)
≥600	26 (65.0)	59 (58.4)
Clinical stage		
IIIa	2 (5.0)	22 (21.8)
IIIb	5 (12.5)	19 (18.8)
IIIc	4 (10.0)	1 (1.0)
IVa	6 (15.0)	50 (49.5)
IVb	23 (57.5)	9 (8.9)
First‐line chemotherapy		
EP	12 (30.0)	40 (39.6)
EC	23 (57.5)	55 (54.5)
Others	5 (12.5)	6 (5.9)
Efficacy of first‐line chemotherapy		
CR	0	0
PR	21 (52.5)	51 (50.5)
SD	15 (37.5)	38 (37.6)
PD	4 (10.0)	12 (11.9)
DCR	36 (90.0)	89 (88.1)
ORR	21 (52.5)	51 (50.5)
First‐line chemotherapy median PFS (months)	6.27 (95% CI, 4.51 to 8.02)	6.97 (95% CI, 5.09 to 8.85)
First‐line PFS (months)		
≤6	18 (45.0)	41 (40.6)
>6	22 (55.0)	60 (59.4)
Radiotherapy before therapy		
Yes	25 (62.5)	55 (54.5)
No	15 (37.5)	46 (45.5)
Brain metastases		
Yes	12 (30.0)	30 (29.7)
No	28 (70.0)	71 (70.3)
Bone metastases		
Yes	13 (32.5)	30 (29.7)
No	27 (67.5)	71 (70.3)
Liver metastases		
Yes	11 (27.5)	22 (21.8)
No	29 (72.5)	79 (78.2)
Performance status^a^		
0	0	5 (5.0)
1	26 (65.0)	96 (95.0)
2	13 (32.5)	0
3	1 (2.5)	0
Serum sodium levels^a^		
Normal	25 (62.5)	88 (87.1)
Hyponatremia	15 (37.5)	13 (12.9)
ICIs in the therapy		
Pembrolizumab	15 (37.5)	0
Nivolumab	14 (35.0)	0
Atezolizumab	11 (27.5)	0
Lines of therapy		
Second‐line	20 (50.0)	101 (100.0)
Third‐line	13 (32.5)	0
Fourth‐line and subsequent‐line	7 (17.5)	0
Tumor cavitation after therapy		
Yes	8 (20.0)	0
No	32 (80.0)	0

Abbreviations: CR, complete response; DCR, disease control rate; EC, etoposide + carboplatin; EP, etoposide + cisplatin; ICIs, immune‐checkpoint inhibitors; ORR, objective response rate; PD, progressive disease; PFS, progression‐free survival; PR, partial response; SD, stable disease.

^a^ Measured at the beginning of therapy.

Among the patients in combination therapy group, 38 (95.0%) were male and 35 (87.5%) were smokers. Patients who received combination therapy had a median age of 64 years (range, 49–77 years). At the time of receiving combination therapy, 5 patients (12.5%), 11 patients (27.5%), 8 patients (20.0%), and 16 patients (40.0%) were at clinical stages of T1, T2, T3, and T4, respectively. In addition, 17 patients (42.5%) were at N2 stage while 23 patients (57.5%) were at N3 stage. Moreover, 11 patients (27.5%) were at M0 stage while 29 patients (72.5%) were at M1 stage (Table 1). Overall, 11 patients (27.5%) were at clinical stage III, whereas 29 patients (72.5%) were at clinical stage IV. Before combination therapy, 10 patients received chest radiotherapy, 6 patients received radiotherapy for distant metastatic lesions, while 9 patients received radiotherapy for both chest and distant metastatic lesions.

Overall, 40 patients in the combination therapy group had failed prior chemotherapy. Twelve patients (30.0%) received an etoposide plus cisplatin (EP) regimen, 23 patients (57.5%) received an etoposide plus carboplatin (EC) regimen, while 5 patients (12.5%) received other regimens as first‐line treatments. Responses to first‐line treatments and PFS are shown in Table 1.

At diagnosis, the PD‐L1 tumor proportion score (TPS) for tumor cells (DACO PD‐L1 IHC 22C3 pharmDx) in 19 patients (47.5%) in the combination therapy group was assessed. Eighteen patients were PD‐L1 negative while 1 was PD‐L1 TPS positive (PDL1 staining 8%).

Anlotinib was orally administered once a day (10 mg) from days 1 to 14 of a 21‐day cycle. Among the 40 patients in the combination therapy group, 15 (37.5%) were treated with pembrolizumab, 14 (35.0%) were treated with nivolumab, while the other 11 (27.5%) were treated with atezolizumab.

In the chemotherapy group, docetaxel (750 mg/m^2^ of body surface area) was intravenously administered every 3–4 weeks until disease progression, discontinuation due to toxicity or other reasons.

Anlotinib plus PD‐1/PD‐L1 blockade therapy was used as second‐line therapeutic option for 20 patients (50.0%), third‐line therapy for 13 patients (32.5%), and fourth‐line and subsequent therapy for 7 patients (17.5%).

There were no patients who had received immunotherapy or antiangiogenic agents before the administration of anlotinib and PD‐1/PD‐L1 inhibitor combination regimen.

### 3.2 Efficacy and safety

The median follow‐up time for all patients was 22.13 months (95% CI, 20.29 to 23.98).

In terms of BORs, compared with the paclitaxel monotherapy group, the combination therapy group showed better efficacies; 8 patients (20.0%) had PD, 26 (65.0%) had SD, 6 (15.0%) had a PR, and none had a CR (*p* = 0.014). The ORRs of the combination therapy and paclitaxel monotherapy groups were 15.0% and 8.9% (*p* = 0.451), respectively. The DCRs of the combination therapy and paclitaxel monotherapy groups were 80.0% and 54.5% (*p* = 0.005), respectively (Table 2).

**TABLE 2:** Relapse and survival data for the anlotinib and PD‐1/PD‐L1 inhibitors combination therapy and paclitaxel monotherapy groups

Observed indicators	Anlotinib and PD‐1/PD‐L1 inhibitors combination therapy (*N* = 40)	Paclitaxel monotherapy (*N* = 101)	*p*‐value
BOR			0.014
CR	0	0	
PR	6 (15.0%)	9 (8.9%)	
SD	26 (65.0%)	46 (45.5%)	
PD	8 (20%)	46 (45.5%)	
ORR	15.0%	8.9%	
DCR	80.0%	54.5%	
Relapse			
Median PFS	3.40 months (95% CI, 2.75–4.05)	2.83 months (95% CI, 2.02–3.64)	0.022
6‐month PFS rate	27.7%	10.1%	
12‐month PFS rate	16.6%	4.5%	
Survival			
Median OS	8.20 months (95% CI, 4.03–12.38)	5.87 months (95% CI, 4.60–7.14)	0.048
6‐month OS rate	57.8%	45.5%	
12‐month OS rate	36.1%	23.4%	

Abbreviations: CR, complete response; DCR, disease control rate; ORR, objective response rate; OS, overall survival; PD, progressive disease; PFS, progression‐free survival; PR, partial response; SD, stable disease.

At last follow‐up visit, 30 patients (75.0%) in the combination therapy group and 97 patients (96.0%) in paclitaxel monotherapy group had discontinued treatments. In the combination therapy group, 28 cases were discontinued due to disease progression and 2 cases due to adverse reactions.

**FIGURE 2:**
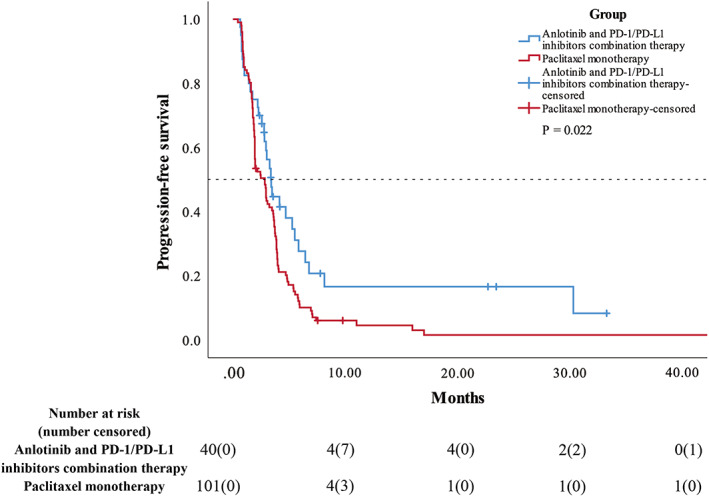
Kaplan–Meier curves showing PFS of anlotinib and PD‐1/PD‐L1 inhibitors combination therapy group vs paclitaxel monotherapy group. PFS, progression‐free survival.

The median PFS for the combination therapy and paclitaxel monotherapy groups were 3.40 months (95% CI, 2.75–4.05) and 2.83 months (95% CI, 2.02–3.64), respectively, with *p =* 0.022 (Figure 2). The 6‐month and 12‐month PFS rates were 27.7% and 16.6%, respectively, for the combination therapy group, and 10.1% and 4.5%, respectively, for the paclitaxel monotherapy group.

**FIGURE 3:**
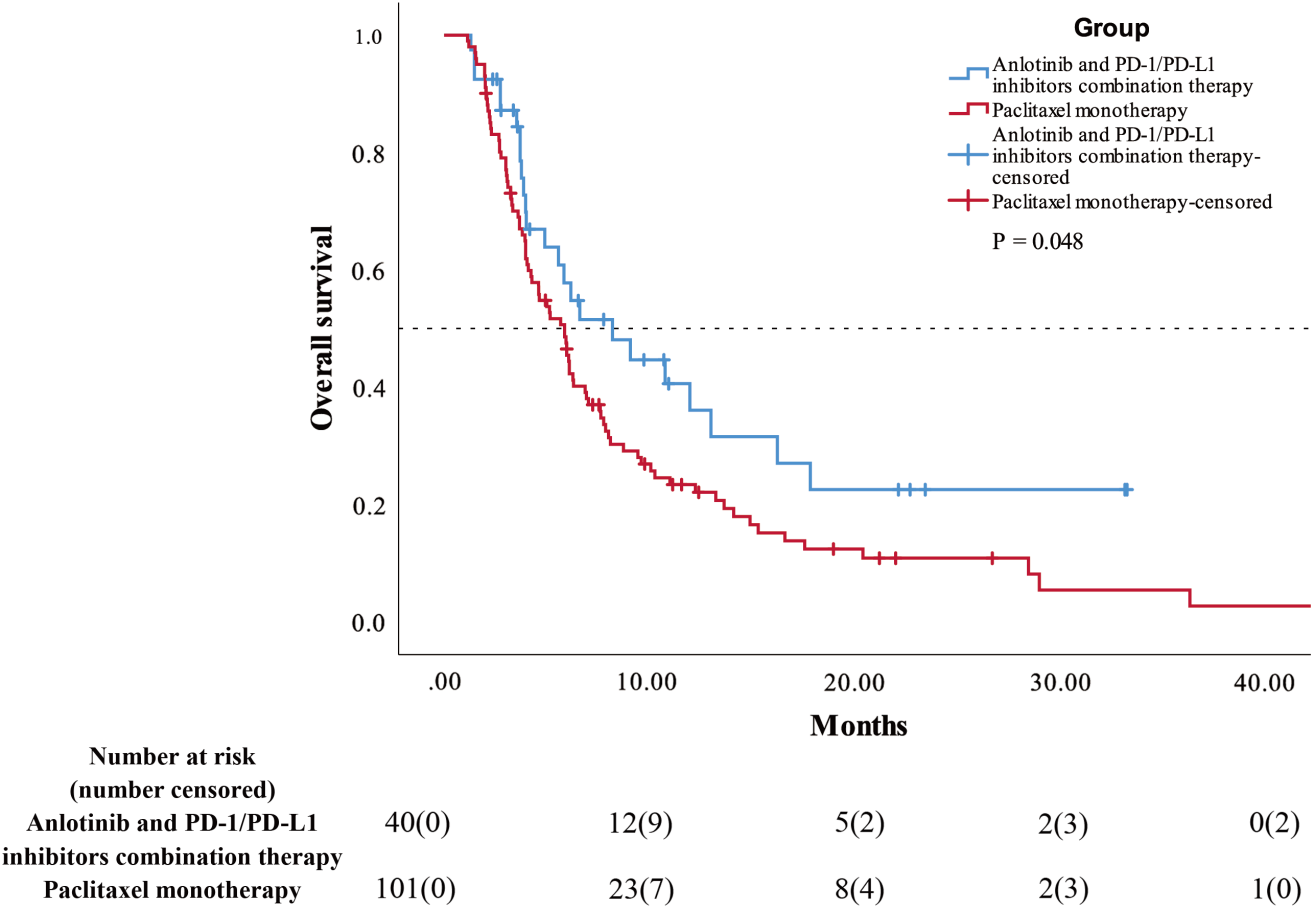
Kaplan–Meier curves showing OS of anlotinib and PD‐1/PD‐L1 inhibitors combination therapy group vs paclitaxel monotherapy group. OS, overall survival.

At last follow‐up visit, 24 patients (60.0%) in the combination therapy group had died, compared to 86 patients (85.1%) in the paclitaxel monotherapy group. Their median OS were 8.20 months (95% CI, 4.03–12.38) and 5.87 months (95% CI, 4.60–7.14), respectively, with *p* = 0.048 (Figure 3). The 6‐month and 12‐month OS rates were 57.8% and 36.1%, respectively, for the combination therapy group, and 45.5% and 23.4%, respectively, for the paclitaxel monotherapy group (Table 2). Some of the advanced SCLC patients who were still taking the anlotinib and PD‐1/PD‐L1 inhibitors combination regimen had long‐lasting benefits, with their PFS exceeding 20 months or even 33 months.

Two patients discontinued the combination therapy due to anorexia (grade 3) and fatigue (grade 3). Twenty‐five patients in the combination therapy group exhibited AEs such as hypertension, hepatic dysfunction, hypothyroidism, and oral ulcers, however, the AEs were manageable and reversible (grades 1–2) (Supplementary Material). The most common multitargeted antiangiogenic TKI therapy‐related AEs were hypertension (15.0%), anorexia (10.0%), and fatigue (10.0%). The most common multitargeted antiangiogenic TKI‐ or ICIs‐related AEs were hypothyroidism (15.0%) and hepatic dysfunction (15.0%). No grade 4 or 5 AEs were reported.

### 3.3 Subgroup analysis of combination therapy group

In the combination therapy group, there were five never‐smokers. Three of the five never‐smokers showed no disease progression, and two had died at last follow‐up. The median PFS for never‐smokers was not achieved. The median OS for never‐smokers was 9.07 months (95% CI, 1.51–16.63). For smokers, the median PFS and OS were 3.37 months (95% CI, 2.77–3.96) and 6.60 months (95% CI, 3.26–9.94), respectively. Differences between the median OS for never‐smokers and smokers were insignificant (*p* = 0.369).

At the beginning of combination therapy, 12 patients were diagnosed with brain metastases; 6 of whom had received cranial radiotherapy. After combination therapy, 3 patients had PD, 8 had SD, and 1 had a PR. They showed a median PFS of 2.80 months (95% CI, 0.00–5.69) and median OS of 5.83 months (95% CI, 0.00–15.82), respectively. Patients without brain metastases had a median PFS and OS of 3.40 months (95% CI, 3.08–3.72) and 8.20 months (95% CI, 4.03–12.37), respectively. Differences in median OS for patients with and without brain metastases were insignificant (*p* = 0.797).

Fifteen patients (37.5%) were diagnosed with hyponatremia while 25 patients (62.5%) were diagnosed with normal serum sodium levels. The median PFS for patients with hyponatremia and normal serum sodium levels were 3.40 months (95% CI, 1.42–5.38) and 3.37 months (95% CI, 2.72–4.02), respectively. The median OS for patients with hyponatremia and normal serum sodium levels were 11.97 months (95% CI, 1.00–22.93) and 8.20 months (95% CI, 4.16–12.24), respectively, with a *p*‐value of 0.996.

In the combination therapy group, 22 patients achieved a PFS of more than 6 months from the first‐line chemotherapy, while 18 patients achieved a PFS of no more than 6 months. The median PFS and OS for patients who achieved better treatment outcomes from prior chemotherapy were 5.27 months (95% CI, 1.74–8.79) and 10.77 months (95% CI, 6.67–14.86) with combination therapy, respectively. The median PFS and OS of the patients who failed to achieve a good PFS from prior chemotherapy were 2.93 months (95% CI, 1.99–3.88) and 6.17 months (95% CI, 2.84–9.49) with combination therapy, respectively. The median OS of patients with a better duration of response to prior chemotherapy was longer than that of patients with a shorter duration of response to prior chemotherapy (median OS 10.77 months vs. 6.17 months). However, the difference did not reach statistical significance (*p* = 0.256). The treatment outcomes from prior chemotherapy do not seem to influence the efficacy of combination therapy.

Patients who received anlotinib and PD‐1/PD‐L1 inhibitors combination therapy as the second‐line therapeutic option had a median PFS of 4.70 months (95% CI, 2.31–7.10) and a median OS of 6.60 months (95% CI, 0.24–12.96). For patients who received the combination therapy as the third‐line or subsequent therapeutic option, the median PFS and OS were 2.93 months (95% CI, 2.64–3.23) and 8.20 months (95% CI, 2.71–13.69), respectively. Differences in median OS between patients who received combination therapy as second‐line or subsequent therapies were insignificant (*p* = 0.742).

**FIGURE 4:**
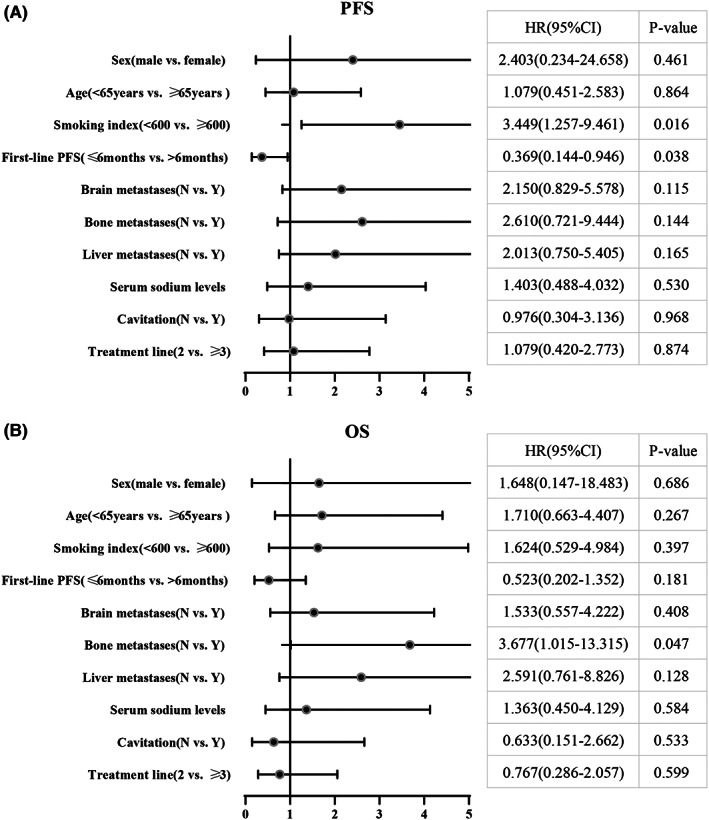
Multivariate Cox regression related to (A) PFS and (B) OS of anlotinib and PD‐1/PD‐L1 inhibitors combination therapy group. PFS, progression‐free survival; OS, overall survival.

The multivariate Cox regression analysis related to PFS and OS of the observation group is shown in Figure 4.

## 4 DISCUSSION

This study evaluated the efficacy and safety of combination therapy using the multitargeted angiogenesis inhibitor (anlotinib) combined with immunotherapeutic PD‐1/PD‐L1 inhibitors as second‐line and subsequent treatment options for advanced SCLC patients. Compared with paclitaxel monotherapy as a second‐line therapy, patients treated with this combined regimen had improved clinical outcomes with good therapeutic efficacies and tolerable AEs.

Chemotherapy is still the main second‐line treatment option for a majority of ED‐SCLC patients; however, treatment outcomes are dismal due to its modest efficacy and significant hematologic toxicity. The single‐agent regimen of standard cytotoxic agents, including paclitaxel, docetaxel, gemcitabine, and vinorelbine have been studied in some phase II clinical trials as second‐line therapies, with modest outcomes.^25^ A randomized phase III trial showed that when used as the second‐line treatment option for ED‐SCLC, topotecan resulted in a median PFS of 3.5 months and a median OS of 7.8 months.^11^ The quest for more effective therapeutic approaches has not abated.

Although immunotherapy plays a key role in treatment of solid tumors, evidence suggests that ICIs are more likely to benefit patients with tobacco exposure and a high mutation burden. In the same breath, ICIs have been hypothesized to be beneficial in SCLC patients.^26^ However, PD‐1 blockade monotherapy did not show OS benefits for SCLC when used as the second‐line or later‐setting therapeutic option, which may be due to insufficient lymphocyte infiltrations in SCLC.^26^, ^27^ The median OS for patients treated with nivolumab as the second‐line or subsequent therapeutic option were 5.6 months and 7.5 months in CheckMate 032 and CheckMate 331, respectively.^12^, ^28^ The median PFS and OS for patients treated with pembrolizumab as the third‐line treatment option were 2.0 months and 7.7 months, respectively, in KEYNOTE‐028 and KEYNOTE‐158.^13^ ICIs monotherapy has not shown compelling clinical benefits in ED‐SCLC.

The initial attempt to add the antiangiogenic agent (bevacizumab) to standard chemotherapy in previously untreated ED‐SCLC (SALUTE Trial) did not result in OS benefits.^29^ Thus, antiangiogenesis therapy had not been further explored and applied in SCLC until the small‐molecule multitargeted angiogenesis inhibitor (anlotinib) showed a survival advantage in clinical trials. As a third‐line therapy and late‐line treatment option, anlotinib monotherapy in ED‐SCLC (ALTER 1202) achieved a median PFS of 4.1 months and a median OS of 7.3 months.^30^

The effects of multitargeted angiogenesis inhibitors are multifaceted, including the promotion of vascular maturation and normalization.^31^ The combination of multitargeted angiogenesis inhibitors and ICIs can confer more significant synergistic therapeutic benefits than monotherapy.^32^ There is a complex relationship between tumor angiogenesis and the tumor immune microenvironment. Tumors alter the differentiation of DCs, leading to a reduction in the number of mature DCs, impairing antigen presentation by DCs, and preventing T‐cell activation via VEGF. This results in an immunosuppressive microenvironment.^33^ High levels of VEGF can also increase the number and proliferation of immunosuppressive cells, such as Treg cells, MDSCs, and M2‐like TAMs.^34^ Antiangiogenic drugs can reprogram pro‐tumor M2‐like TAMs toward an anticancer M1 phenotype, increase the number of DCs and TAMs, and improve antigen presentation. Anlotinib, a novel multitargeted TKI, strongly inhibits VEGFR, FGFR, PDGFR, and c‐kit, acting on both the TME and the tumor itself.^14^ Anlotinib can increase the infiltrations of innate immune cells, including NK, and APC, but decrease the number of M2‐like TAMs.^18^ Anlotinib was shown to increase tumor vascular vessel formation, at least partially, by preventing the early exhaustion of CD4+ T cells.^35^ ICIs suppress the activities of immunosuppressive cells and indirectly downregulate the expressions of angiogenic factors, thereby improving tumor vascular vessel formation.^36^ A combination of anlotinib with a PD‐1 checkpoint inhibitor reversed the immunosuppression caused by upregulated PD‐L1, leading to prolonged vascular normalization.^35^ The incorporation of antiangiogenic therapy into immunotherapy enhanced the efficacy of both agents and transformed the immunosuppressive tumor microenvironment into an immunosupportive one. Some clinical trials have investigated the efficacy of this combination regimen in various solid tumors.^22^, ^23^, ^37^

In this study, the combination of anlotinib with PD‐1/PD‐L1 inhibitors had a median OS of 8.20 months in only 50% of patients receiving it as the third‐line therapy or subsequent therapy, even as an end‐stage treatment. Some patients can benefit from this combination therapy for a long time. In the real world, most of the ED‐SCLC patients who accepted anlotinib combined with ICIs could not tolerate chemotherapy. These patients may suffer and have a relatively poor prognosis after other conventional treatments.

At last follow‐up visit, the six patients who achieved a BOR of a PR had promising clinical benefits with a median PFS of 30.30 months and none of them died. The SCLC tumor shrinkage rate was positively correlated with PFS and OS. These findings are in tandem with those reported by Fujii M.^38^

Studies have also reported that never‐smokers have better survival rates than smokers,^39^, ^40^ which is inconsistent with our finding. In this study, differences between the median OS of never‐smokers and smokers (9.07 months vs. 6.60 months, *p* = 0.369) were insignificant. SCLC is a high‐mutation–burden cancer, and non‐smokers might have different disease genotypes and phenotypes. However, our study did not explore the genomic profile and tumor mutational burden of never‐smokers and smokers. In our previous study, the most frequent mutated genes in non‐smokers diagnosed with ED‐SCLC were TP53 (100%), RB1 (73%), SMAD4 (35%), KDR (23%), NOTCH1 (20%), and PTEN (20%).^41^

The efficacy of immunotherapy such as pembrolizumab was not satisfactory in lung cancer patients who received late‐line therapy, compared with patients who received ICIs as first‐line therapy; this may be due to immunocompromised and impaired T‐cell production and function after disease progression.^42^ However, when used as a third‐line or late‐line therapy in ED‐SCLC, the combination of ICIs and antiangiogenic agents resulted in a similar OS rate, compared with its use as a second‐line therapy. Therefore, the combination of ICIs and antiangiogenic TKIs could confer synergistic therapeutic benefits and even benefit immunocompromised ED‐SCLC patients when used as a late‐line therapy.

This combination did not result in a significant survival benefit for patients with brain or liver metastases or cavitated lesions. There is a need to identify appropriate biomarkers to guide the use of this combination treatment.

Regarding AEs, the safety profile was generally consistent with known safety profiles of either ICIs or anlotinib alone and other combinations of PD‐L1 inhibitor plus antiangiogenic agents. No new AEs were identified. During treatment with the combination regimen, two patients changed treatment regimens because of severe fatigue and anorexia, leading to drug discontinuation. Although anorexia, fatigue, hypothyroidism, hepatic dysfunction, and hypertension occurred in no less than 10% of patients in the combination group, the AEs were manageable in most patients, with a low incidence of grade 3 adverse events, all of which recovered after intervention or discontinuation. This is consistent with findings from previous studies: antiangiogenic agents and ICI have a synergistic anticancer effect and may reduce the risk of toxicity.^43^

The main limitation of this study is related to the lack of a scientifically rigorous cohort design and the existence of selection bias due to its retrospective nature. Hence, prospective clinical trials, involving relatively large sample sizes, are advocated to verify the feasibility of this treatment option. T‐cell subset analysis was not performed in this study. Studies should aim at identifying biomarkers for predicting the prognosis of patients. Only anlotinib is currently available in China, however, it may be a good representative of the other multitargeted antiangiogenic TKIs.

## 5 CONCLUSION

In conclusion, anlotinib plus PD‐1/PD‐L1 blockade therapy has shown significant benefits as a second‐line and later‐line therapeutic option for advanced SCLC without causing more serious adverse reactions than chemotherapy. The combination in ED‐SCLC warrants further clinical verification.

## AUTHOR CONTRIBUTIONS

**Lian Yu:** Data curation (lead); formal analysis (lead); investigation (lead); methodology (lead); software (lead); writing – original draft (lead); writing – review and editing (equal). **Jianlin Xu:** Data curation (supporting); formal analysis (supporting); investigation (supporting); methodology (supporting); software (supporting). **Rong Qiao:** Data curation (supporting); investigation (supporting); methodology (supporting). **Baohui Han:** Project administration (supporting); supervision (supporting); validation (supporting); visualization (supporting). **Hua Zhong:** Formal analysis (supporting); resources (supporting); supervision (supporting); validation (supporting); visualization (supporting). **Runbo Zhong:** Conceptualization (lead); data curation (supporting); project administration (lead); resources (lead); supervision (lead); validation (lead); visualization (lead); writing – review and editing (lead).

## CONFLICT OF INTEREST

The authors declare no conflicts of interest.

## ETHICS STATEMENT

This retrospective study was approved by the institutional review board and ethics committee of Shanghai Chest Hospital. The requirement for participants' informed consent was waived.

## Supporting information


Supinfo S1

## Data Availability

The data that support the findings of this study are available from the corresponding author upon reasonable request.
